# Topological band-order transition and quantum spin Hall edge engineering in functionalized X-Bi(111) (X = Ga, In, and Tl) bilayer

**DOI:** 10.1038/srep33395

**Published:** 2016-09-14

**Authors:** Youngjae Kim, Won Seok Yun, J. D. Lee

**Affiliations:** 1 Department of Emerging Materials Science, DGIST, Daegu 42988, Republic of Korea

## Abstract

Functionalized X-Bi bilayers (X = Ga, In, and Tl) with halogens bonded on their both sides have been recently claimed to be the giant topological insulators due to the strong band inversion strengths. Employing the first-principles electronic structure calculation, we find the topological band order transition from the order p – p – s of the X-Bi bilayers with halogens on their both sides to the new order p – s – p of the bilayers (especially for X = Ga and In) with halogen on one side and hydrogen on the other side, where the asymmetric hydrogen bonding simulates the substrate. We further find that the p – s bulk band gap of the bilayer bearing the new order p – s – p sensitively depends on the electric field, which enables a meaningful engineering of the quantum spin Hall edge state by controlling the external electric field.

Topological insulators (TIs) have the insulating bulk state and simultaneously the spin-polarized gapless edge (or surface) state. The edge states are topologically protected against nonmagnetic impurities. Compared to the three-dimensional (3D) TIs, however, it is well known that the edge states of the two-dimensional (2D) TIs are more robustly protected due to the forbidden backscattering, which makes the 2D TIs attract much attention. 2D TIs, also known as the quantum spin Hall insulators (QSHIs)^1^,^2^,^3^, therefore, would be highly promising in the field of spintronics or other low-power-consuming technologies due to the dissipationless transport channels driven by the forbidden backscattering process on their edges.

There are kinds of low-dimensional TIs; quantum well (QW) structures like CdTe/HgTe/CdTe^4^ and GaAs/Ge/GaAs^5^ and 2D TIs like Sn^6^,^7^, Ge^8^, Bi^9^,^10^,^11^, Sb^12^,^13^ and Si^8^,^14^. QW structures have a very small band gap so that they cannot realize the topologically protected order at the room temperature, whereas 2D TIs show the potential for the room temperature operation due to large bulk band gaps. More recently, it was found that the alloy-type bilayer of group III elements with Bi would have a huge nontrivial band gap (~1 eV), i.e., the giant topological insulator, and thus retain a superior survivability of the edge states at high temperatures and a controllable electronic structure with chemically diverse functionalization^15^,^16^. Although the halogenated bilayer of X-Bi (X = group III elements like Ga, In, and Tl), for instance, the chlorinated gallium bismuth (Cl-GaBi-Cl), could be considered in a freestanding state within the computational treatment^17^, it must be grown on a suitable substrate with the covalent-*σ* bonds between the bilayer and substrate atoms for a practical realization^18^,^19^. In fact, it has been demonstrated that few-layered 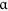-Sn (001) should be grown on InSb (001), which has the gapless band dispersion on the surface^20^, and also 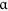-Sn (111) (stanene) be grown on InSb (111) substrate^21^,^22^. Therefore, it is vital to study the electronic structure reconstruction resulted from an interaction between the bilayer and the substrate beyond the electronic structure of the freestanding state.

In the present study, using the first-principles electronic structure calculation, we investigate the electronic structure reconstruction from the X-Bi bilayer (X = Ga and In) with halogens on their both sides to the bilayer with halogen on one side and hydrogen on the other side. Hydrogen bondings simulate the substrate effects and identify the quantum spin Hall phase of the 2D materials more efficiently without real substrate calculation^18^,^19^,^23^. In this consideration, we find the topological band order transition from the order p – p – s of the former to the new order p – s – p of the latter. In the new order p – s – p, the p – s bulk band gap is found to sensitively depend on the external electric field, which changes the Dirac bands and makes it possible to control the quantum spin Hall edge transport with respect to the electric field. Finally, it is confirmed that the fluorinated GaBi grown on the In-terminated InSb (111) or the Cd-terminated CdTe (111) substrate should have the electronic structure with the band order p – s – p.

## Results and Discussion

**Figure 1:**
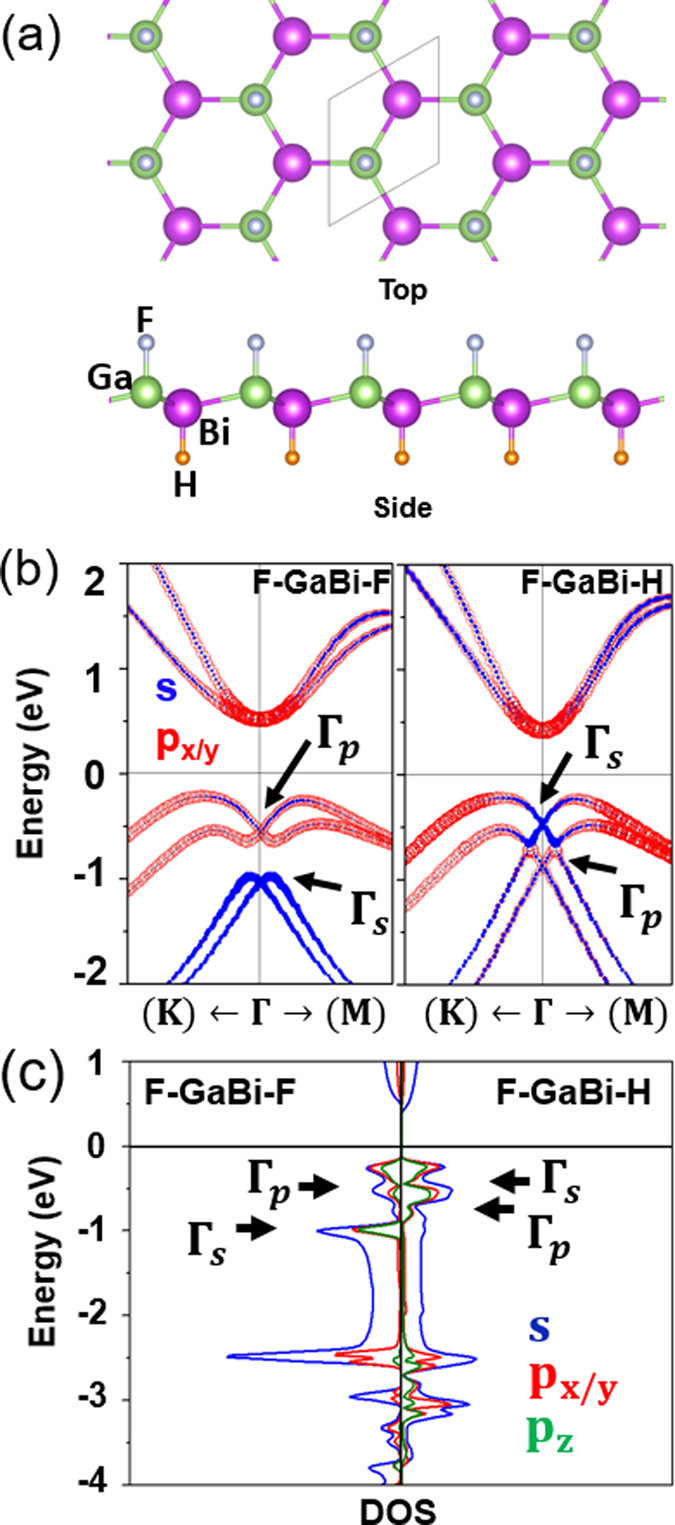
(**a**) Crystal structures - top and side views of F-Ga-Bi-H. White, green, purple, and orange balls represent F, Ga, Bi, and H atoms, respectively. (**b**) Electronic band structures of F-GaBi-F and F-GaBi-H. s bands are designated by blue closed circles and p bands by red open circles. The sizes of circles imply the orbital band weights. (**c**) Partial density of states of s, p_x/y_, and p_z_ bands from Ga and Bi for F-GaBi-F and F-GaBi-H.

We consider the honeycomb crystal structure of the functionalized X-Bi (111) layer, i.e., Y-X-Bi-H (Y = halogens like F, Cl, Br, and I) (in Fig. 1(a)). For all the combinations, performing the first-principles electronic structure calculation, the fully relaxed lattice constants are obtained (see Table S1) as 4.71, 4.64, 4.63, and 4.62 Å for F-GaBi-H, Cl-GaBi-H, Br-GaBi-H, and I-GaBi-H, respectively, 5.02, 4.94, 4.92, and 4.91 Å for F-InBi-H, Cl-InBi-H, Br-InBi-H, and I-InBi-H, respectively, and 5.19, 5.11, 5.07, and 5.06 Å for F-TlBi-H, Cl-TlBi-H, Br-TlBi-H, and I-TlBi-H, respectively. It is noted that the lattice parameters are decreased when X-Bi bilayers are halogenated from F to I, which is found to be consistent with the halogenated stanene^6^. It is further noted that GaBi and TlBi with the hydrogen bondings on the Bi side have lower total energy than those on the Ga or Tl side (see Table S1) from which an assumption of the Bi-substrate bonding is preferred as an energetically stable structure.

The band positions at the Γ point near the Fermi level have been calculated for Y-GaBi-H, Y-InBi-H, and Y-TlBi-H using the generalized gradient approximation (GGA) without or with the spin-orbit (SO) coupling (see Fig. S1). It has been reported previously that, in the GGA + SO calculation, Y-GaBi-Y, Y-InBi-Y^16^,^24^, and Y-TlBi-Y^16^ have the band order p – p – s. In contrast to that, Y-GaBi-H and Y-InBi-H is found to exhibit the inverted band order p – s – p at the Γ point, undergoing the band order transition from Y-GaBi-Y and Y-InBi-Y. Nevertheless, let us note that there is no band order transition in Y-TlBi-H differently from Y-GaBi-H or Y-InBi-H, as shown in Fig. S1(c).

Nontrivial band order transitions are confirmed in the adiabatic continuation approach^25^. The GGA p orbitals which are degenerate at the Fermi level are split when the SO coupling turns on and the band gaps are opened. Generally, the nontrivial topological phase will not be destructed even when each X or Bi side is functionalized to have chemical bonds with different kinds of atoms. To demonstrate the band order transition occurring from F-GaBi-F to F-GaBi-H, the electronic band structures and the partial densities of states (PDOS) of the two systems are provided in Fig. 1(b,c). The s orbital at the Γ point, which is designated as Γ_s_, is positioned at ~ −1 eV for F-GaBi-F, whereas the s orbital shifts upward up to ~ −0.5 eV due to the asymmetric hydrogen bondings and a concomitant decrease of the lattice parameters for F-GaBi-H because the s orbital is strongly hybridized with p_x/y_ and p_z_ orbitals (sp^3^ hybridization). According to Fig. S2, the same band order transition is observed from F-InBi-F to F-InBi-H, whereas, for the case of TlBi, Γ_s_ is positioned relatively deep so that the band order p – p – s does not change even from F-TlBi-F to F-TlBi-H. In addition, we present the band structures of Y-X-Bi-H and Y-X-Bi-F with or without the SO coupling in Fig. S3. One can see that the s orbital in TlBi does not take a part in the sp^3^ hybridization in Figs S2 and S3.

**Figure 2:**
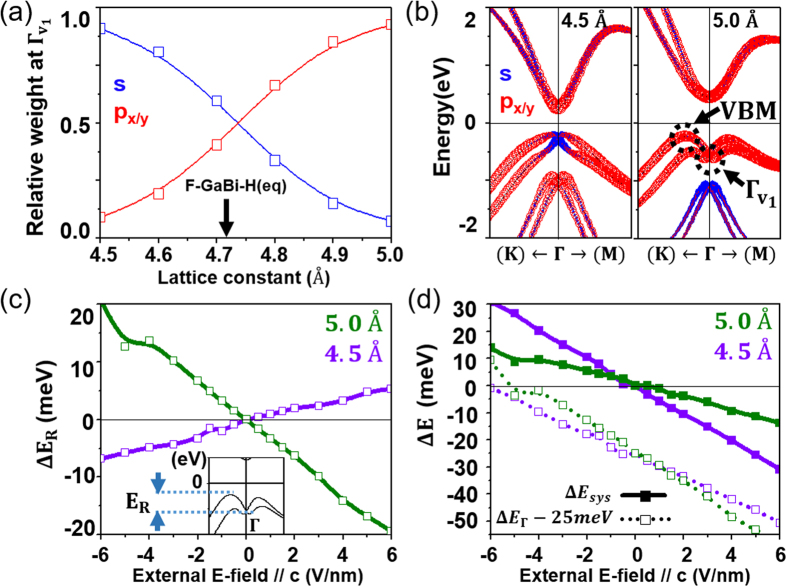
(**a**) Relative weights of the s and p orbitals at Γ_v1_ as a function of the lattice parameters of the tensilely or compressively strained F-GaBi-H. A black arrow indicates the equilibrium F-GaBi-H (4.71 Å). (**b**) Electronic band structures of the strained F-GaBi-H. In the figure, 4.5 Å and 5.0 Å represent F-GaBi-H whose lattice constants are suppressed to 4.5 Å and elongated to 5.0 Å, respectively. (**c**) Changes of Rashba strengths (E_R_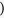 from the equilibrium F-GaBi-H are given with respect to the applied electric field along the c direction. 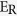 is defined as the inset, i.e., an energy difference between VBM and Γ_v1_. (**d**) Changes of the system gap ΔΕ_sys_ and the Γ-point gap ΔE_Γ_ from the equilibrium F-GaBi-H are given with respect to the applied electric field along the c direction.

Figure S4(a) shows that the strain effect can determine the magnitude of an orbital hybridization. In Fig. 2(a), we calculate the relative weight of s and p orbitals contributed to Γ_v1_ in the tensilely or compressively strained F-GaBi-H, where Γ_v1_ is the highest valence state at the Γ point. As the lattice constant increases (the tensile strain is applied), the relative weight makes a crossover from the s-majority to the p-majority at about +0.8% strain (i.e., corresponding to 4.75 Å). The competition between those orbital contributions can eventually lead to the band order transition as shown in Fig. S5(a). Hence it is understood that the system exhibits the different band symmetries at the two extremes, say at the lattice constants of 4.5 Å and 5.0 Å. The lattice constant of 4.5 Å, where Γ_v1_ is mainly composed of s orbitals, gives the p – s bulk band gap, but that of 5.0 Å gives the p – p bulk band gap. In Fig. 2(b) (See more details in Fig. S6 (a)), this is confirmed in the electronic structures of F-GaBi-H with the lattice constants 4.5 Å and 5.0 Å, where the valence band maxima (VBM) are in fact dominated by s orbitals and p orbitals, respectively. Changes of the Rashba strength E_R_ relative to the zero-field F-GaBi-H, ΔE_R_, are provided at 4.5 Å and 5.0 Å with respect to the external electric field along the c direction in Fig. 2(c). It is interesting to note that ΔE_R_ at 4.5 Å and 5.0 Å varies oppositely with respect to the electric field. Large differences in the behaviors of the system gap change (relative to the zero-field F-GaBi-H) under the external electric field, as shown in Fig. 2(d), are mainly caused by the different Rashba responses. The p orbital (making the p – p bulk band gap) is more sensitive to the SO coupling and its strong Rashba response largely cancels the system band gap change in a case of 5.0 Å. In the context, this could also explain an appreciable change of the system band gap at 4.5 Å. In contrast with the cases of Sb (111) or silicene grown on Bi/Si (111)^12^,^14^, the topological band gap of functionalized GaBi is so big that there is a probability to control the edge states by the external electric field.

**Figure 3:**
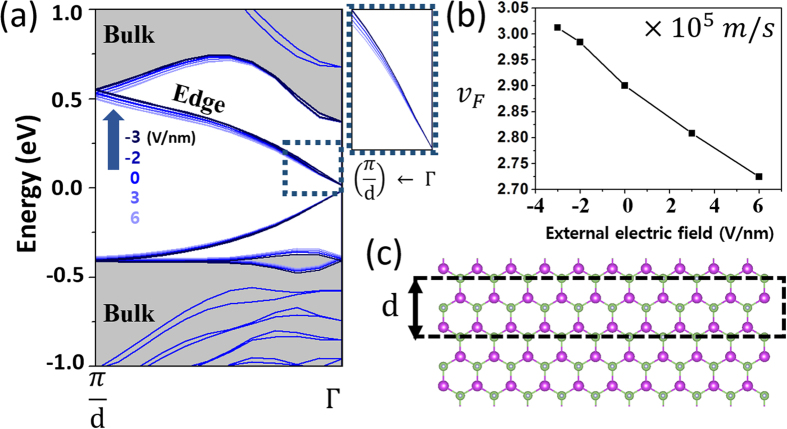
(**a**) Electronic structure of the armchair F-GaBi-H nanoribbon (compressively strained to the lattice constant 4.5 Å) is represented. External electric field induces the topological band order transition and consequently changes the transports of the edge state. The inset (dashed box) shows slopes of the massless Dirac bands near the Fermi level. (**b**) The Fermi velocity of the Dirac spins is manipulated by the external electric field. (**c**) Crystal structure of the armchair F-GaBi-H ribbon with *N* = 20 slabs and the periodicity d = 7.79 Å.

The p – s bulk band gap sensitively responsible under the external electric field would eventually renormalize the quantum spin Hall edge state. We calculate the electronic band structure of an armchair-edged nanoribbon (ANR) F-GaBi-H bilayer (compressively strained to the lattice constant 4.5 Å) with *N* = 20 atoms across the ribbon and identify the edge state in Fig. 3. Clearly, the band structure reveals the helical edge states with massless Dirac nodes at the Γ point inside the bulk band gap. Such an existence of the topologically protected states in the ANR F-GaBi-H is consistent with ANRs of other well-known 2D TIs, i.e. H-GaBi-H and F-GaBi-F in Fig. S7(a,b). Remarkably, according to an application of the external electric field, the slopes of the Dirac bands significantly changes and the Fermi velocity of the Dirac spins, which determines the transport of the spin Hall edge state, varies by ~10% for a change of the electric field, i.e., −2 V/nm to 6 V/nm, as shown in Fig. 3(b). This should realize a practical manipulation of the quantum spin Hall edge transport.

**Figure 4:**
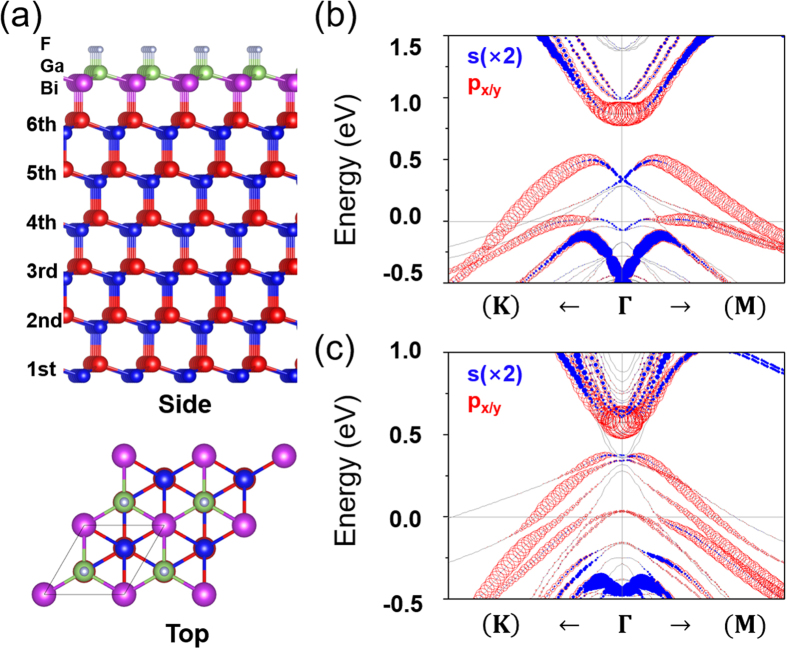
(**a**) Crystal structures (side and top views) of F-GaBi/CdTe(111) or F-GaBi/InSb(111). Red and blue balls indicate Cd (or In) and Te (or Sb), respectively. (**b**) Electronic band structure of F-GaBi/CdTe(111). (**c**) Electronic band structure of F-GaBi/InSb (111). Electronic structure calculations are done with GGA + SO.

As mentioned previously, the asymmetric hydrogen bonding to the Bi-side of GaBi bilayers has been attempted to simulate the substrate effects. We need to confirm this, in particular, whether the band order p – s – p of F-GaBi-H could be surely maintained when F-GaBi is actually grown on a suitable substrate. We consider the Cd-terminated CdTe (111) and the In-terminated InSb (111) for candidate substrates, which have the lattice parameters 4.687 Å^26^ and 4.694 Å^27^, respectively, comparable to the equilibrium F-GaBi-H. As the model for the calculation, we consider six-layer slabs to avoid an interaction between two interfaces in Fig. 4(a). Formation properties of two kinds of substrate-supported fluorinated GaBi’s are first investigated. The binding energy, E_B_ is defined as


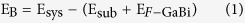


where E_sys_, E_sub_, and E_F-GaBi_ are the total energy of the model system, the energy of an isolated substrate, and the energy of the freestanding F-GaBi without any passivation on the Bi side. The obtained binding energies are −1.74 eV and −2.07 eV for covalently bonded F-GaBi/CdTe (111) and F-GaBi/InSb (111) per unit cell, respectively. Meanwhile, the binding energy between F-GaBi and H (for a freestanding F-GaBi-H) is −3.77 eV so that F-GaBi-H is found more stable in our calculation. Electronic band structures of F-GaBi grown on the two substrates are illustrated in Fig. 4(b,c). Both of two have the metallic bands because the charge transfer is induced by the asymmetric (111) zinc-blende type structure. By the small electron doping, however, it would be enabled that the Fermi level shifts up to the middle of the gap at ~0.5 eV. This consequently makes these systems TIs with the system gaps of 0.38 eV and 0.2 eV for F-GaBi/CdTe (111) and F-GaBi/InSb (111), respectively. According to the full band structures of Fig. S8(a,b), we can recognize the band inversion at the Γ point like a freestanding F-GaBi-H. The p and s orbitals are positioned at the conduction band minimum (CBM) and the highest valence state at the Γ point (i.e., Γ_v1_), respectively. These orbitals make the Γ-point gap (i.e., the energy gap at the Γ point) of the nontrivial bulk band to be 0.53 eV for F-GaBi/CdTe (111) and 0.22 eV for F-GaBi/InSb (111). From Fig. 4, therefore, it is strongly evidenced that the topological band order transition actually occurs from the band order p – p – s of the freestanding F-GaBi-F to the new order p – s – p of F-GaBi grown on CdTe (111) or InSb (111).

## Summary

In summary, we have performed the first-principles electronic structure calculation of Y-X-Bi-H (X = group III elements like Ga, In, and Tl and Y = halogens like F, Cl, Br, and I) to simulate the substrate-supported functionalized bilayers. We have analyzed the topological band order transition from p – p – s to p – s – p occurring in the route from Y-X-Bi-Y to Y-X-Bi-H (especially for X = Ga and In). The *s* orbitals at Γ_v1_ of F-GaBi-H and F-InBi-H are strongly hybridized with the p_x/y_ and p_z_ orbitals so that their energetics become sensitive to the structural deformation, which leads to the band order transition and results in the p – s bulk band gap. The p – s bulk band gap is found highly responsive to the external electric field, which would enable an engineering of the quantum spin Hall edge transport. Finally, we have shown that the asymmetric hydrogen bonding to the Bi-side of F-GaBi could well describe the substrate effects from the calculation for the substrate-supported F-GaBi structure.

## Computational Methods

We employed the first-principles electronic structure calculation performed on Vienna *ab-initio* simulation package (VASP) code with Perdew-Burke-Ernzerhof (PBE) generalized gradient approximation (GGA) exchange correlation functional^28^,^29^,^30^. Freestanding structures are calculated using Monkhorst-Pack^31^ grid of **k**-point 29 × 29 × 1 mesh and substrate-supported calculations are for 15 × 15 × 1 mesh with the projector argumented wave pseudopotential^32^. Energy cutoffs of 550 eV for GaBi, 500 eV for InBi and TlBi are adopted with 10^−4^ eVÅ^−1^ of convergence criterion.

## Additional Information

**How to cite this article**: Kim, Y. *et al*. Topological band-order transition and quantum spin Hall edge engineering in functionalized X-Bi(111) (X = Ga, In, and Tl) bilayer. *Sci. Rep.* **6**, 33395; doi: 10.1038/srep33395 (2016).

## Supplementary Material

Supplementary Information
