# Supplementary material for: Topological band-order transition and quantum spin Hall edge engineering in functionalized X-Bi(111) (X = Ga, In, and Tl) bilayer
Source: Sci Rep. 2016 Sep 14;6:33395. doi: 10.1038/srep33395 (PMC5022058; doi:10.1038/srep33395)
Supplement: Supplementary Information [file srep33395-s1.pdf]

Supplementary Material

Topological band-order transition and quantum spin Hall edge engineering in functionalized X-Bi(111) (X = Ga, In, and Tl) bilayer

Youngjae Kim, Won Seok Yun, and J. D. Lee*

Department of Emerging Materials Science, DGIST, Daegu 42988, Republic of Korea

*To whom correspondence should be addressed. E-mail: jdlee@dgist.ac.kr

We used the density functional theory based on Vienna *ab-initio* simulation package (VASP) code. The generalized gradient approximation (GGA) by the Perdew-Burke-Enzerhoff (PBE) functional and the projector augmented wave (PAW) potentials are used. Cutoff energies of 550 and 500 eV for the freestanding GaBi and In(or Tl)Bi bilayers, respectively, were adopted for the plane-wave expansion and a 29×29×1 Monkhorst-Pack k-point grid used. Atomic positions were fully relaxed until the force on each atom is less than 10^{-4} eV $Å^{-1}$.

Table S1. Calculated lattice constants of functionalized GaBi, InBi, and TlBi and the total energy differences between Y-XBi-H and H-XBi-Y systems. Here, X and Y indicate elements of group IIIA (Ga, In, and Tl) and group VIIA (F, Cl, Br, and I), respectively.

System	Lattice Constant (Å)	$E_{\text{Y-XBi-H}} – E_{\text{H-XBi-Y}}$ (eV)
F-GaBi-F	4.87 [S1]	n/a
Cl-GaBi-Cl	4.79 [S1]	n/a
Br-GaBi-Br	4.78 [S1]	n/a
I-GaBi-I	4.77 [S1]	n/a
F-InBi-F	5.20 [S1]	n/a
Cl-InBi-Cl	5.12 [S1]	n/a
Br-InBi-Br	5.09 [S1]	n/a
I-InBi-I	5.07 [S1]	n/a
F-TlBi-F	5.29	n/a
Cl-TlBi-Cl	5.16	n/a
Br-TlBi-Br	5.13	n/a
I-TlBi-I	5.06	n/a
F-GaBi-H	4.71	-0.674
Cl-GaiBi-H	4.64	-0.501
Br-GaBi-H	4.63	-0.416
I-GaBi-H	4.62	-0.304
F-InBi-H	5.02	n/a
Cl-InBi-H	4.94	n/a
Br-InBi-H	4.92	n/a
I-InBi-H	4.91	n/a
F-TlBi-H	5.19	-0.240
Cl-TlBi-H	5.11	-0.431
Br-TlBi-H	5.07	-0.482
I-TlBi-H	5.06	-0.503

We have considered two types of functionalized XBi bilayers. One is fully halogenated XBi bilayer (Y-XBi-Y) and the other is half-halogenated and half-hydrogenated XBi bilayer (Y-XBi-H or H-XBi-Y) to

simulate the substrate-supported structure. Note that X and Y indicate the elements of group IIIA (Ga, In, and Tl) and group VIIA (F, Cl, Br, and I), respectively. Replacing X by the elements of group IIIA (from Ga to Tl), the lattice constant gets to be larger gradually. In addition, the lattice constant of Y-XBi-H system is always smaller than Y-XBi-Y system. In cases of the substrate-supported situation, there exist two cases: Y-XBi-H and H-XBi-Y. As listed in Table S1, it is clearly seen that Y-XBi-H system have lower total energy than H-XBi-Y system. It is a stable structure that the halogen atoms which have strong electronegativities are adsorbed onto X side and atoms of the substrate bonded to Bi side (i.e, Y-XBi-substrate). Due to the reason, we have focused on Y-XBi-H system.

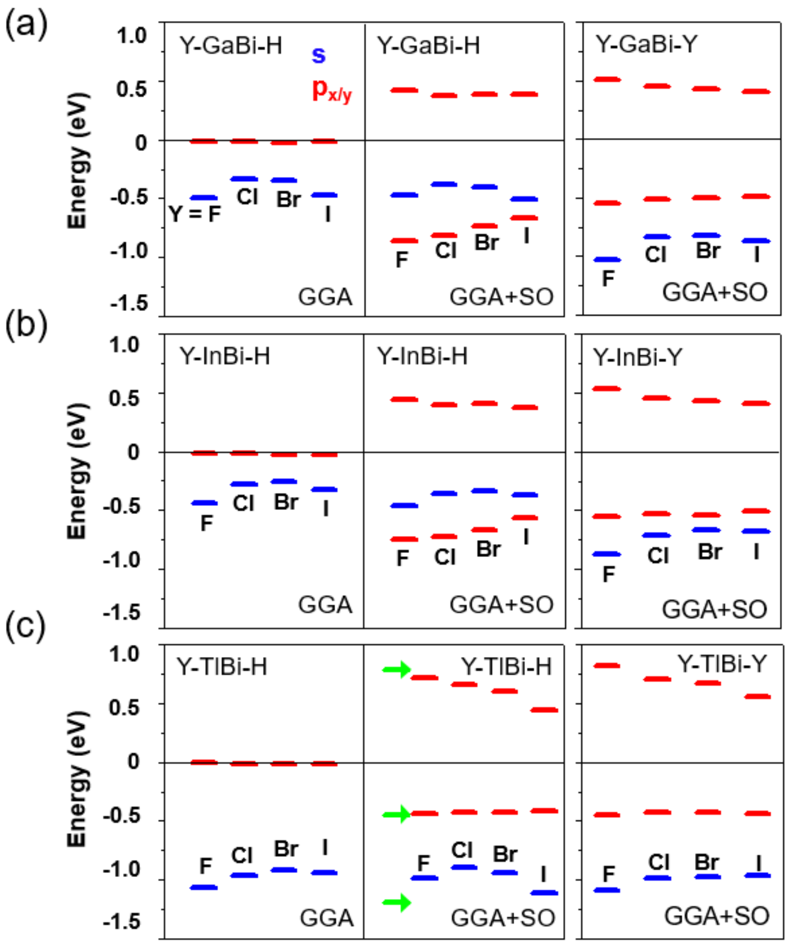

Figure S1. Positions of s (blue) and $p_{x/y}$ (red) bands at the Γ point of (**a**) Y-GaBi-H, (**b**) Y-InBi-H, and (**c**) Y-TlBi-H based on GGA and GGA+SO. Green arrows indicate the s or p band positions calculated by HSE06.

In Fig. S1, the band positions at the Γ point near the Fermi level have been calculated for Y-GaBi-H, Y-InBi-H, and Y-TlBi-H using the GGA without or with SO. In the GGA+SO calculation, Y-GaBi-Y, Y-InBi-Y, and Y-TlBi-Y have the band order p – p – s. In contrast to that, as shown in Fig. S1(a,b), Y-GaBi-H and Y-InBi-H exhibit the inverted band order p – s – p at the Γ point, which undergoes the band order transition from Y-GaBi-Y and Y-InBi-Y. Nevertheless, note that there is no band order transition in Y-TlBi-H due to the dehybridized s orbital, being distinct from Y-GaBi-H and Y-InBi-H.

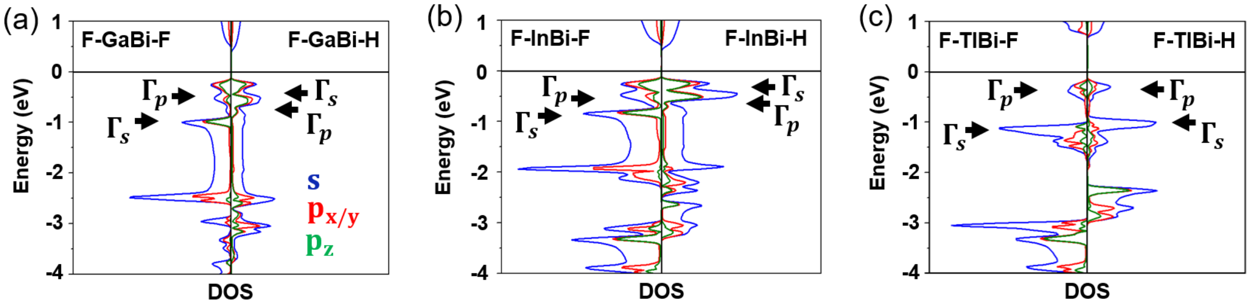

Figure S2. Partial density of states of s, $p_{x/y}$, and p_z bands from (**a**) Ga and Bi for F-GaBi-F and F-GaBi-H, (**b**) In and Bi for F-InBi-F and F-InBi-H, (**c**) Tl and Bi for F-TlBi-F and F-TlBi-H.

To demonstrate the band order transition occurring from F-X-Bi-F to F-X-Bi-H with X = Ga and In, the partial densities of states (PDOS) of s, $p_{x/y}$, and p_z orbitals are provided in Fig. S2(a,b). The s orbital at the Γ point, which is designated as Γ_s, is positioned at ~ -1 eV for F-X-Bi-F, whereas the s orbital shifts upward up to ~ -0.5 eV due to the asymmetric hydrogen bondings and a concomitant decrease of the lattice parameters for F-X-Bi-H. For the case of TlBi, on the other hand, as displayed in Fig. S2(c), Γ_s is positioned relatively deep due to the strong band inversion strength so that the band order p – p – s does not change even from F-TlBi-F to F-TlBi-H. Furthermore, Fig. S2(c) shows that the s orbital (near ~ -1 eV) at the Γ point is highly localized and core-like, which hinders the hybridization with p orbitals. X-Bi (111) bilayers form the buckled honeycomb structure that is supported by the sp^3-like s-p hybridization. The s orbitals near the Γ point in GaBi and InBi are strongly hybridized with p orbitals and also respond sensitively to the

surface chemical effects accompanying structural changes. For TlBi, however, the s-p hybridized states are positioned at ~ -2 eV far below the Fermi level. Even if those states are strongly affected by the structural change from F-TlBi-F to F-TlBi-H, the states cannot drive the band order transition.

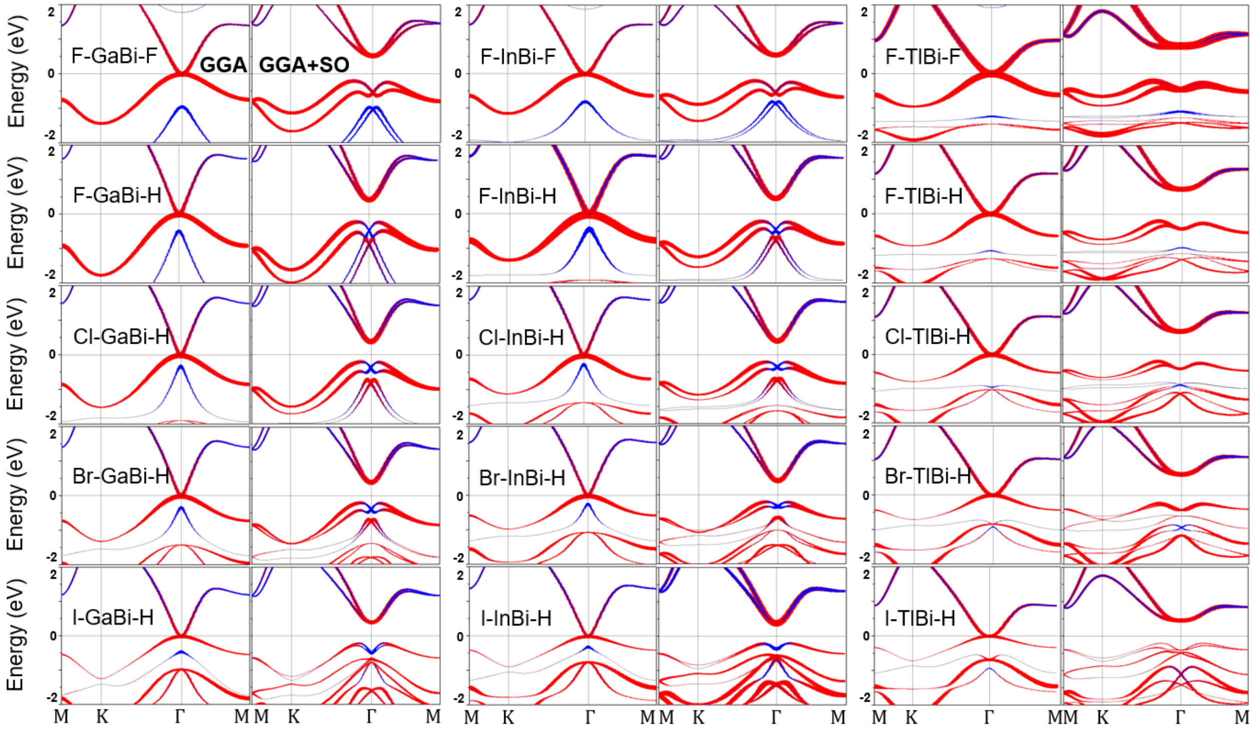

Figure S3. Calculated band structures of both Y-XBi-Y and Y-XBi-H systems using GGA and GGA+SO. Red and blue colors indicate p and s orbitals, respectively.

In Fig. S3, we represent the calculated band structures of F-XBi-F and Y-XBi-H systems. All of Y-XBi-H systems with equilibrium lattice constants have nontrivial band structures according to GGA results. The s orbital at the Γ point in TlBi is shown to be a strongly flat and localized band, in contrast to GaBi or InBi case. Therefore, the s orbital in TlBi does not take a part in the sp^3 hybridization so that it would be insensitive to the structural deformation.

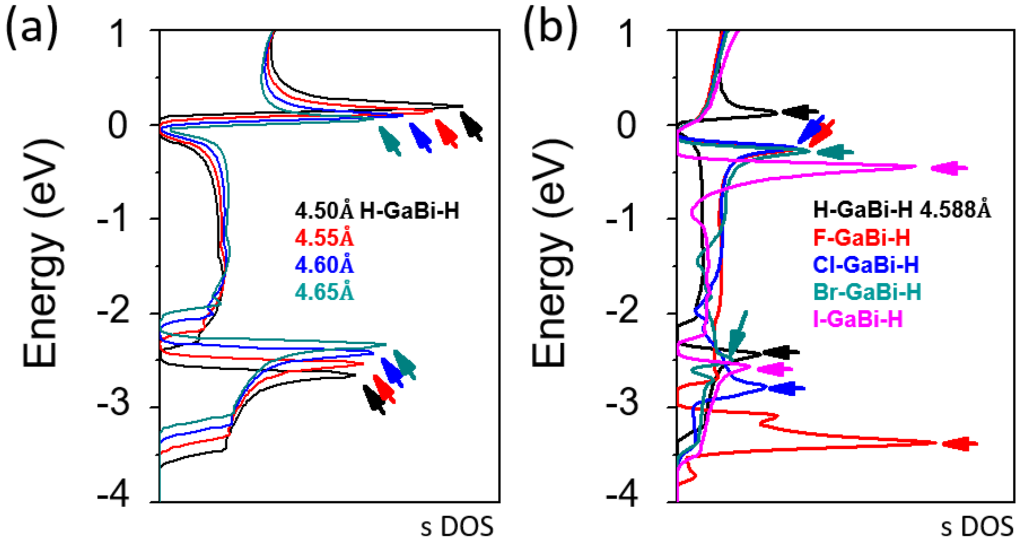

Figure S4. Calculated density of states (DOS) for the s orbitals of Ga and Bi in the functionalized GaBi bilayer without SO coupling using GGA method. (**a**) DOS for the s orbitals in H-GaBi-H system as a function of various lattice constants. (**b**) DOS for the s orbitals in H-GaBi-H and Y-GaBi-H systems at fixed lattice constant of 4.588 Å. In (**a**) and (**b**), each arrow indicates the van Hove singularities of s orbitals in the electronic structure.

It is well known that halogenated X-Bi bilayers have intrinsically nontrivial band structures without SO. In order to understand the trivial/nontrivial phase transition, we calculated the density of states (DOS) for the s orbitals in H-GaBi-H and Y-GaBi-H systems, as shown in Fig. S4. In Fig. S4(a), increasing its lattice constant from 4.50 Å to 4.65 Å, the s orbital singularities above the Fermi level are energetically pulled down, whereas those below the Fermi level pushed up. Considering that the large lattice constant suppresses the orbital hybridization, the s orbital above the Fermi level has an odd parity and that below the Fermi level a bonding state with even parity. With SO turned on, the odd s state is occupied due to the band inversion. In Fig. S4(b), particularly, Y-GaBi-H system with lattice constant of 4.588 Å (;the equilibrium lattice constant of H-GaBi-H [S2]) shows an intrinsically nontrivial band structure without SO because the odd s orbitals are below the Fermi level (i.e., fully occupied). Due to the strong band inversion strength by

halogenation, SO originated from Bi atoms splits degenerated p orbitals and opens a giant gap, i.e., causing the giant topological insulator.

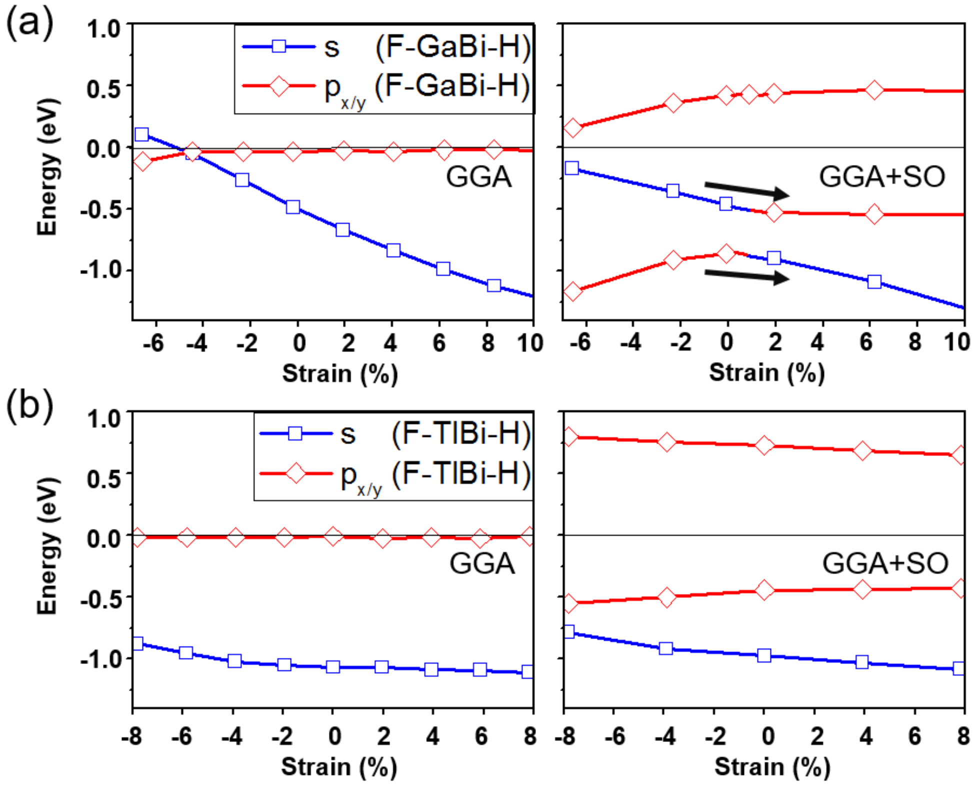

Figure S5. Positions of s and p bands at the Γ point as a function of the applied strain for (**a**) F-GaBi-H and (**b**) F-TlBi-H. Calculations are done with GGA or GGA+SO.

The positions of Γ_s and Γ_p of F-GaBi-H and F-TlBi-H are displayed as a function of the applied strain, which are calculated by GGA and GGA+SO. In Fig. S5(a), according to GGA + SO, Γ_s appears more sensitive to the strain rather than Γ_p, which makes a transition to the band order p – p – s at a small positive strain even if the ground state at the zero strain has the order p – s – p. However, F-TlBi-H is simply not the case. In Fig. S5(b), it is evident that F-TlBi-H is a p – p – s topological insulator throughout a range of the strain given in the figure due to the dehybridized s orbital at ~ -1 eV. This helps F-TlBi-H an advantage of a large band gap over a wide range of the strain.

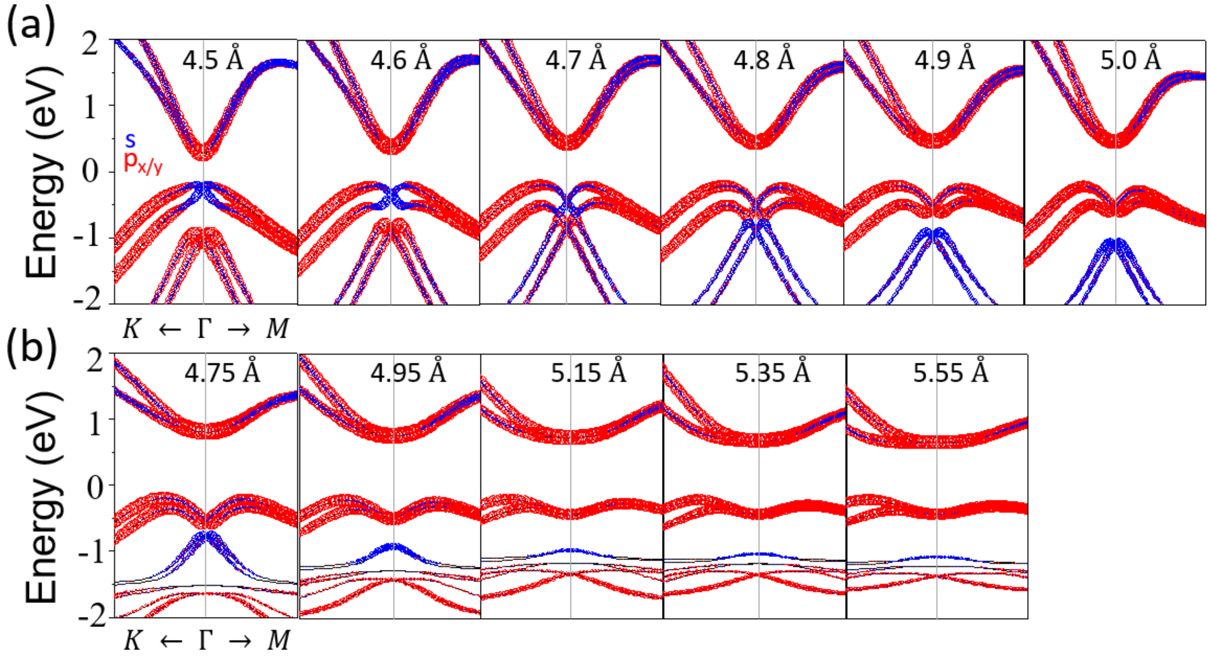

Figure S6. Calculated band structures of (**a**) F-GaBi-H and (**b**) F-TlBi-H systems using GGA+SO as a function of various lattice constants.

Due to the sp^3 type of hybridization, the structural deformation significantly affects the s orbital in F-GaBi-H system. It is understood that the lattice constants of two-dimensional films grown on the substrate could be altered by an interaction with the underlying substrate lattice. As shown in Fig. S5(a), for the 4.5 Å F-GaBi-H system (negatively strained to 4.5 Å), the system band gap consists of p and s orbitals. When the lattice constant increases, the s orbital is gradually replaced by the p orbital. The change of band order from p – p – s to p – s – p occur between 4.7 and 4.8 Å. From the equilibrium lattice F-GaBi-H system (i.e., 4.71 Å), the positive and negative strains selectively result in p – p – s and p – s – p band order, respectively. However, in case of F-TlBi-H system, there is no band order transition (see Fig. S6(b)).

In Fig.S7, the edge states of the fully relaxed H-GaBi-H and F-GaBi-F and the strained F-GaBi-H (same as Fig.3(a) of the main text) assuming the armchair nanoribbons (N = 20) are demonstrated. In Fig. S8,

the full band structures of both F-GaBi/CdTe(111) and F-GaBi/InSb(111) are provided, which are basically same as Fig. 4 of the main text.

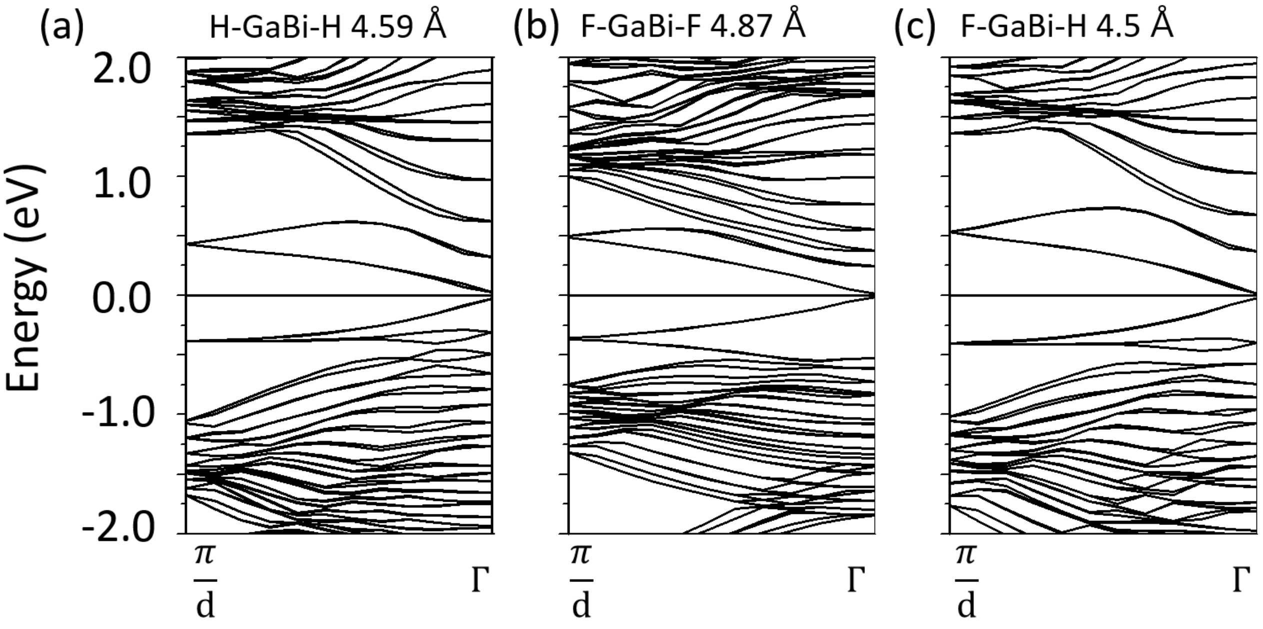

Figure S7. Calculated band structures of (a) the fully relaxed H-GaBi-H with a lattice constant of 4.59 Å, (b) F-GaBi-F (4.87 Å) and (c) the strained F-GaBi-H (4.5 Å) with the geometry of N=20 armchair nanoribbons, respectively.

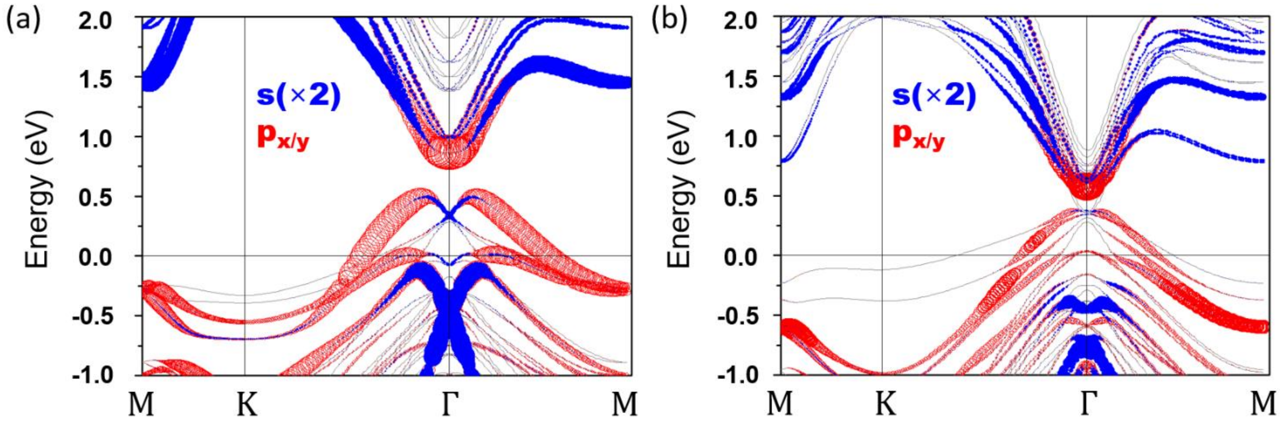

Figure S8. (a) Full band structures of F-GaBi/CdTe(111) and (b) F-GaBi/InSb(111). Calculations are done with GGA+SO.

References

S1. Freitas, R. R. Q. *et al.* Tuning band inversion symmetry of buckled III-Bi sheets by halogenation. *Nanotechnol*. **27**, 055704 (2016).

S2. Ma, Y. *et al.* Two-dimensional inversion-asymmetric topological insulators in functionalized III-Bi bilayers. *Phys. Rev. B* **91,** 235306 (2015).
